# An unusual case of intercostal muscle flap ossification mimicking an intrathoracic rib

**DOI:** 10.1259/bjrcr.20150469

**Published:** 2016-11-02

**Authors:** Sharif Abdullah, Ming Hao Guo, Gail Darling, Demetris Patsios

**Affiliations:** ^1^Joint Department of Medical Imaging, University Health Network, Toronto, Canada; ^2^Faculty of Medicine, University Toronto, Toronto, Canada; ^3^Thoracic surgery, University Health Network, Toronto, Canada

## Abstract

We present a unique case of intercostal muscle flap (ICMF) ossification mimicking an intrathoracic rib diagnosed 3 years after oesophageal perforation repair. A 58-year-old male presented with complaints of mild chest discomfort. Three years ago he had undergone left thoracotomy and primary repair of post-emetic oesophageal perforation. An ICMF had been used to strengthen the repair. Chest X-ray identified a linear calcific density within the left hemithorax. Subsequent thoracic CT characterized the anomaly as ossification of the ICMF. The lesion had the appearance of a well-differentiated intrathoracic rib coursing through the left lower lobe. We discuss the typical appearances of ossified ICMFs and the potential complications resulting from this ossification.

## Summary

Although ossification of intercostal muscle flaps (ICMFs) has been reported in the literature,^1–3^ we were unable to find reports of ossification resembling an intrathoracic rib coursing through the lung. Such ossification can have a variety of appearances on CT and can result in complications such as anastomotic stricture and ischaemia. Surgical technique has been shown to determine whether an ICMF ossifies.^4^

## Clinical presentation

Three years before the present findings, a 58-year-old male had been referred to the thoracic surgery department at Toronto General Hospital with acute onset of post-emetic sharp central back pain. On admission the patient’s vital signs showed tachycardia, hypotension with a systolic blood pressure of 90 mmHg and a fever of 38.4 °C.

On thoracic CT angiogram performed to exclude aortic dissection, free air was identified in the posterior mediastinum adjacent to the distal oesophagus. Subsequent Gastrografin oesophagography revealed a small contained leak at the distal oesophagus. Left thoracotomy was performed. When entering the chest the left sixth intercostal muscle bundle was mobilised and after primary repair of the perforation this ICMF was sutured over the repair acting as a buttress.

The patient recovered well post-operatively and was discharged after 12 days. The initial post-operative follow-up was uneventful. Nevertheless, 3 years later, the patient was referred back to the thoracic surgery clinic by his family doctor because of mild chest discomfort.

## Imaging findings

**Figure 1.**
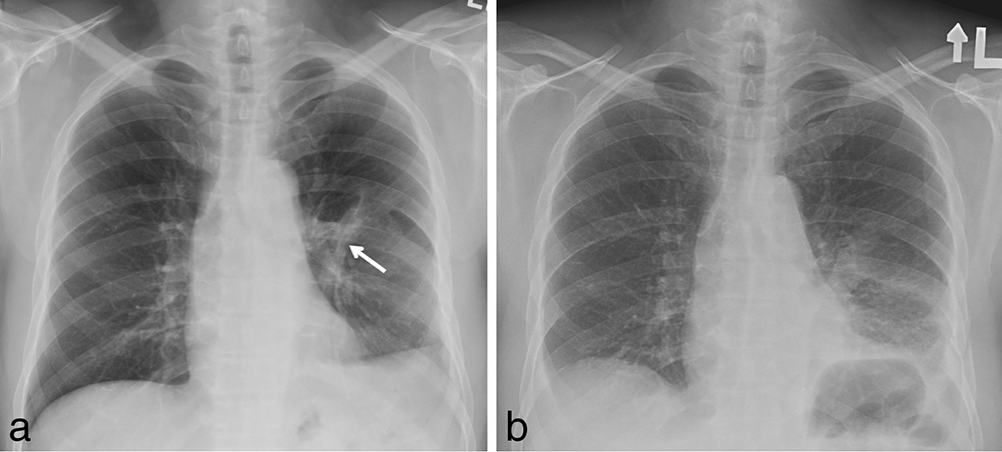
(a) Chest radiograph showing a linear calcific density in the left hemithorax representing the ossified component of the ICMF. (b) Comparison chest radiograph performed 2 years earlier demonstrating absence of the calcific density.

**Figure 2.**
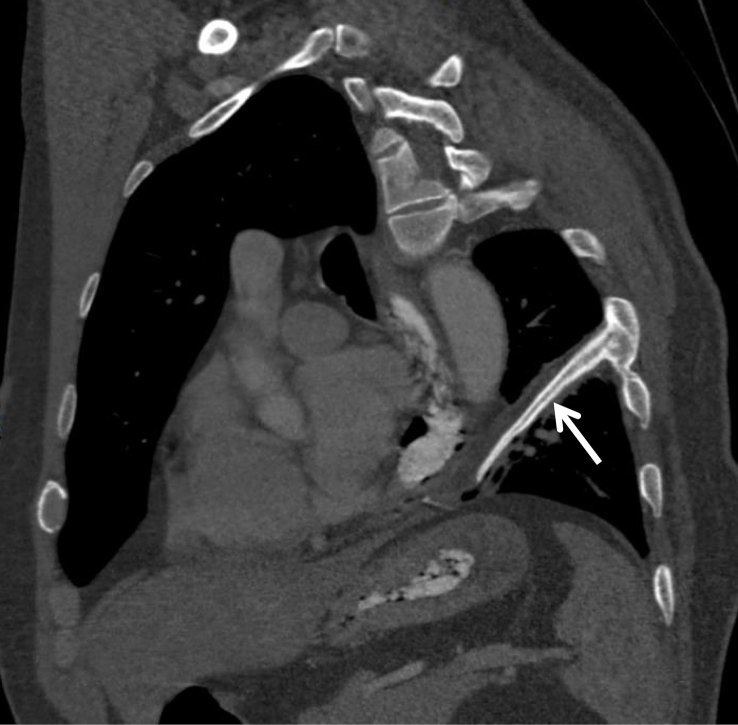
Left anterior oblique reformatted CT image demonstrating the ICMF arising from the left sixth intercostal space. Central ossification resembling a rib is shown.

**Figure 3.**
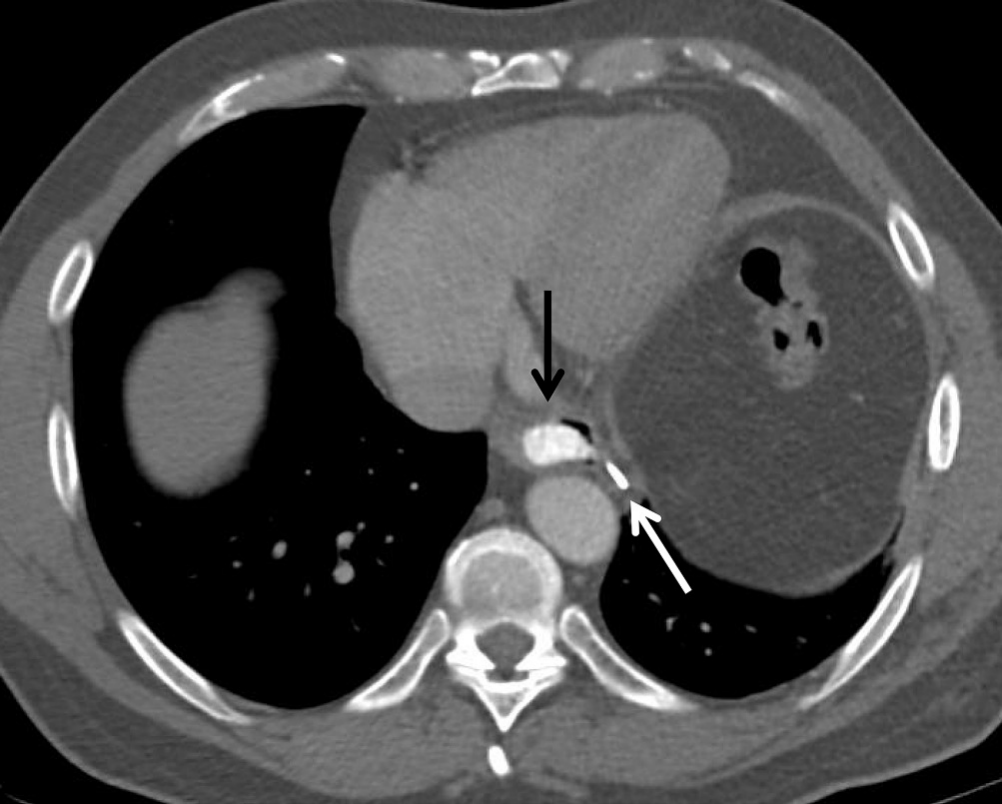
Axial CT image demonstrating a patent oesophagus containing oral contrast (black arrow) at the level of the anastomosis between the distal oesophagus and the ICMF. A surgical staple is shown at the site of anastomosis (white arrow).

**Figure 4.**
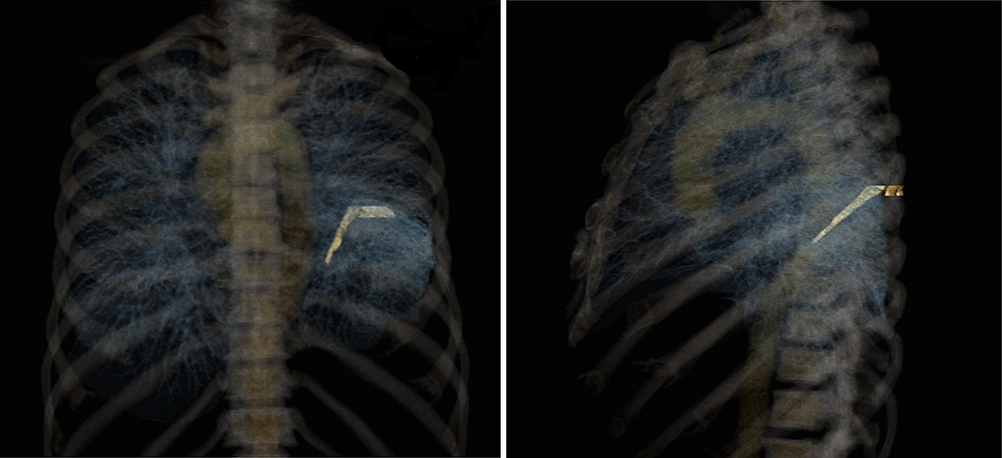
Three-dimensional volume rendered CT images demonstrating the ossified component of the intercostal muscle flap.

**Figure 5.**
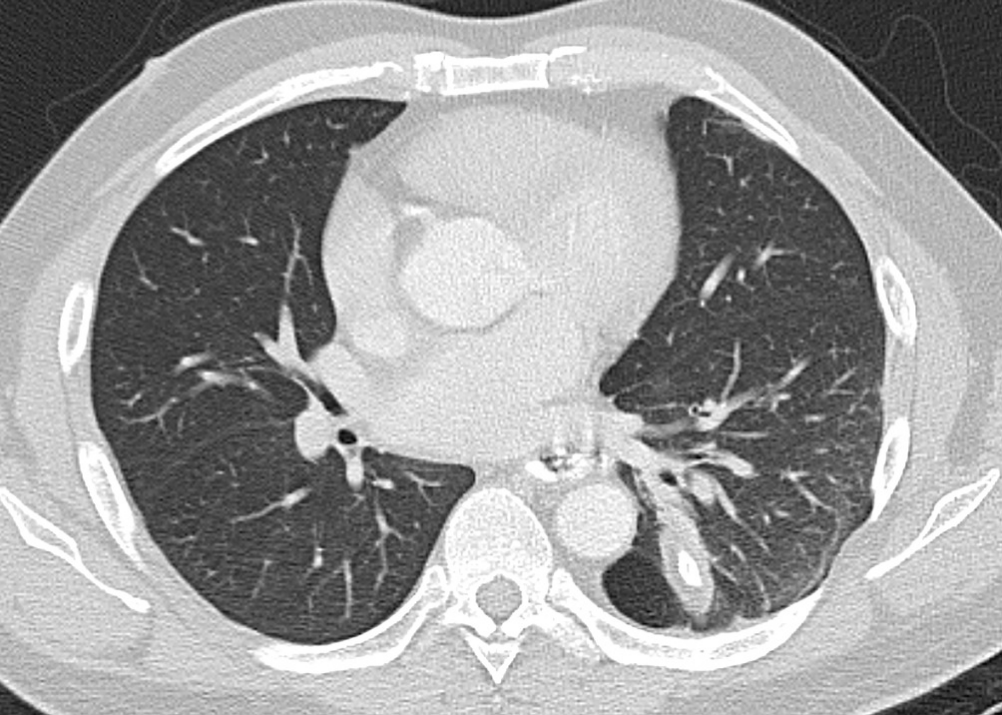
Axial CT image demonstrating the position of the ossified intercostal muscle flap within the left lower lobe.

Chest radiograph was first performed and showed a linear calcific density within a band of soft tissue density in the left hemithorax (Figure 1a). Comparison with a previous chest radiograph (Figure 1b) revealed that this calcification had developed within a 2-year interval. Subsequent thoracic CT demonstrated two continuous parallel linear stripes of calcific density with a lower density stripe in between, coursing along the length of the ICMF (Figures 2 and 3) and mimicking an intrathoracic rib (Figure 4). Interestingly, this structure coursed directly through the left lower lobe, encased in the parietal pleura (Figure 5). The described ossification was largely soldering the posterior arch of the sixth rib (Figure 2). There was no evidence of oesophageal stenosis.

## Treatment and follow-up

The patient’s mild discomfort did not hinder the activities of his daily life. He was discharged from the thoracic surgery clinic with the advice to return should his symptoms worsen.

## Discussion

Post-emetic oesophageal perforation is uncommon and has a high mortality rate if untreated. Primary repair with the use of a pedicled ICMF as a buttress has been described in the literature, as local oedema, necrosis and inflammation could compromise the site of primary repair.^5^ Compared with pleural or pericardial flaps, which have limited vasculature, the ICMF is easily mobilized within the thoracic space and its potential for neovascularisation ensures long-term viability.^5–8^ The use of ICMFs is also indicated in the repair of tracheoesophageal fistulas, buttressing of tracheal and bronchial anastomoses, repair of bronchopleural fistulas and for reconstruction post intrathoracic cancer resection.^9–11^

The ICMF is composed of intercostal vessels, nerves, skeletal muscle and frequently periosteum of the adjacent ribs, which includes pluripotent mesenchymal cells that have the potential to develop into osteoprogenitor cells.^1^ Therefore, calcification and ossification of the muscle flap is a known post-operative phenomenon.^10^ Different surgical techniques for the preparation of ICMFs have been associated with varying degrees of ossification post-operatively. In most studies, periosteum from both adjacent ribs is harvested together with the ICMF. On follow-up, the majority of these exhibited some degree of calcification or ossification.^2,10^ Cerfolio et al^4^ described a technique in which the ICMF was harvested utilising cautery instead of the subperiosteal approach for bronchial buttressing and none of the 301 cases described at 6-month follow-up exhibited any ossification.

Kwek et al^2^ performed a retrospective review on the radiological records of 23 patients in whom an ICMF was utilised for bronchial stump reinforcement post lung resection. At follow-up, all ICMFs showed discontinuous linear calcifications either appearing as a single narrow stripe resembling a surgical staple line, or as two parallel stripes with a lower attenuation central portion, resembling a rib. Calcification was shown as early as 1 week post-operatively and reached a maximal density at a mean of 5 months.

Ossification has been reported to cause bronchial anastomotic stricture or tracheal stenosis if the ICMF is wrapped circumferentially around the underlying structure.^1,3^ If this technique has been utilised, bronchial anastomotic stricture should be excluded on post-operative CT scans. A consequence of this complication is recurrent pneumonia of the affected lung or lobe. If the underlying structure is the oesophagus, stenosis can be excluded on barium oesophagography or thoracic CT with oral contrast.

This case illustrates ICMF ossification post oesophageal perforation repair. On CT, ossification of the flap in this patient resembled an intrathoracic rib arising from the posterior left sixth intercostal space, coursing through the left lower lobe to the distal oesophagus.

## Learning points

1. Ossification of ICMFs is a recognised phenomenon when the periosteum of adjacent ribs is removed together with the flap.
2. This can have several appearances on CT, the most common being a single narrow high-density stripe within the flap or two parallel high-density stripes with a lower density central portion (mimicking a bone).
3. Ossification of ICMFs is usually an incidental finding with no clinical consequences.
4. If the ICMF is wrapped around a bronchus, the trachea or the oesophagus, ossification may result in a stricture, which should be excluded on imaging.

## Consent

Written informed consent was obtained from the patient for publication of this case report, including accompanying images.
